# The Debate on the Ego-Depletion Effect: Evidence from Meta-Analysis with the *p*-Uniform Method

**DOI:** 10.3389/fpsyg.2017.00197

**Published:** 2017-02-14

**Authors:** Desirée Blázquez, Juan Botella, Manuel Suero

**Affiliations:** Methodology and Social Psychology, Universidad Autónoma de MadridMadrid, Spain

**Keywords:** ego-depletion, publication bias, meta-analysis, only significant meta-analysis, *p*-uniform

The crisis of confidence in the field of psychology has to do mainly with the low reliability of the process of collecting and assessing the evidence (Pashler and Wagenmakers, 2012). Questionable practices of researchers, publication bias, and other effects can distort our interpretation of a body of evidence. Each method and technique of analysis has weaknesses and potential threats. The most convincing scenario is one in which a variety of methods with different weaknesses converge on the same conclusion.

Such convergence does not show up for the so-called *ego-depletion effect* (Baumeister et al., 1998). Primary studies using different experimental paradigms have provided inconsistent evidence (e.g., see Kurzban et al., 2013, and the subsequent debate). The first meta-analysis that synthesized the evidence concluded that the effect exists (Hagger et al., 2010). Subsequent meta-analyses with different inclusion/exclusion criteria and specific methods to address the effects of publication bias have concluded that the effect exists, but the estimated effect size (*ES*) is substantially lower (Carter and McCullough, 2014), or even null (Carter et al., 2015). A Registered Replication Reports (RRR) initiative has questioned the replicability (and the very existence) of the ego-depletion effect (Hagger and Chatzisarantis, 2016).

Let us review the results of the meta-analyses. Hagger et al. (2010) collected 198 independent estimates, and their overall *ES* estimate is *d* = 0.62 (0.57–0.67; *I*^2^ = 34.7). Later, Carter and McCullough (2014) argued that the evidence in this field of research can be strongly influenced by small-study effects. Such effects, essentially magnified by the publication bias toward significant results, may have yielded an overestimate of the *ES*, which could in fact be zero. Most procedures for detecting publication bias are based on departures from symmetry in the funnel plot (Light and Pillemer, 1984). However, although publication bias results in such asymmetries, not all asymmetries are a by-product of publication bias. Other factors (e.g., *p-hacking*) can yield indistinguishable effects.

While the asymmetries only call attention to probable *ES* over-estimations, there are also some methods for assessing and correcting the effect of publication bias. Carter and McCullough (2014) applied several of those methods to the dataset of Hagger et al. (2010), including the trim-and-fill procedure (Duval and Tweedie, 2000) and two regression methods proposed by Egger et al. (1997) and refined by Moreno et al. (2009) and Stanley and Doucouliagos (2014). Their corrected estimates for Hagger et al.'s dataset are *d* = 0.48 (trim-and-fill) and *d* = −0.10 or *d* = 0.25 (with the two regression procedures). In their response, Hagger and Chatzisarantis (2014) allege that in those re-analyses, Carter and McCullough (2014) assume the unsupported belief that the asymmetries are due to publication bias and not to other factors.

In a new series of meta-analyses, Carter et al. (2015) proposed deep changes in the inclusion/exclusion criteria and made a more exhaustive search for non-published studies. Their new dataset had 116 independent estimates, 28 (24.14%) in common with those used by Hagger et al. Their overall *ES* estimate was 0.43 (0.34–0.52; *I*^2^ = 71.55), ranging from 0.24 to 0.79, when the studies were disaggregated according to the experimental task. The overall estimate is smaller than that of Hagger et al. but the heterogeneity of the effects (*I*^2^) is much larger.

At first glance, the logical conclusions from that meta-analytic evidence must be that the first meta-analysis overestimated the *ES*, that the actual population *ES* is substantially lower or even null, and that the estimates obtained are most probably by-products of publication bias and other circumstantial factors. We add to this puzzle a re-analysis of the datasets of the two meta-analyses (Hagger et al., 2010; Carter et al., 2015) using the *p*-uniform method. We will show that in the ego-depletion case the conclusions can be seriously influenced by the method chosen to synthesize the evidence (see also Cunningham and Baumeister, 2016).

## Re-analysis with the *p*-uniform method

A recent alternative strategy to address publication bias focuses only on the primary studies with significant results. When a body of research has a noticeable influence of publication bias, the published significant studies better represent the population of all potential significant studies than the non-significant published studies represent the population of non-significant studies. Therefore, inferences made from the significant studies are more reliable than those based on all published studies. The only assumption is that within the statistically significant studies, the conditional probability of being published is independent of the *p*-value. A statistical model of the significant *p*-values under such an assumption (*p*-curve) is used to model the data (Simonsohn et al., 2014), and an estimate of the parameter is obtained for the best fit to a uniform distribution of the normalized values (Van Assen et al., 2015).

Following Simonsohn et al. (2014), Carter and McCullough (2014, footnote 3) explicitly dismissed the *p*-uniform procedure for their meta-analysis arguing that in some primary studies of ego-depletion the basis for significance is an interaction test, instead of the simple effect synthesized in the meta-analysis. Their rationale is that categorization of studies according to the significance of the interaction does not match the categorization based on significance of the simple effect. When both categorizations match, there is no problem. When the interaction is significant but the simple effect is not, that study can be excluded from the dataset before the *p*-uniform analysis. However, the studies with a non-significant interaction but with a significant simple effect can end in the “file-drawer.” As this process would probably eliminate more small- than large-simple effects, the results with the *p*-uniform test would bias the *ES* estimate upwards. Despite that, and according to the recommendations of van Aert et al. (2016), we believe that a *p*-uniform analysis can be validly applied to these datasets.

**Figure 1:**
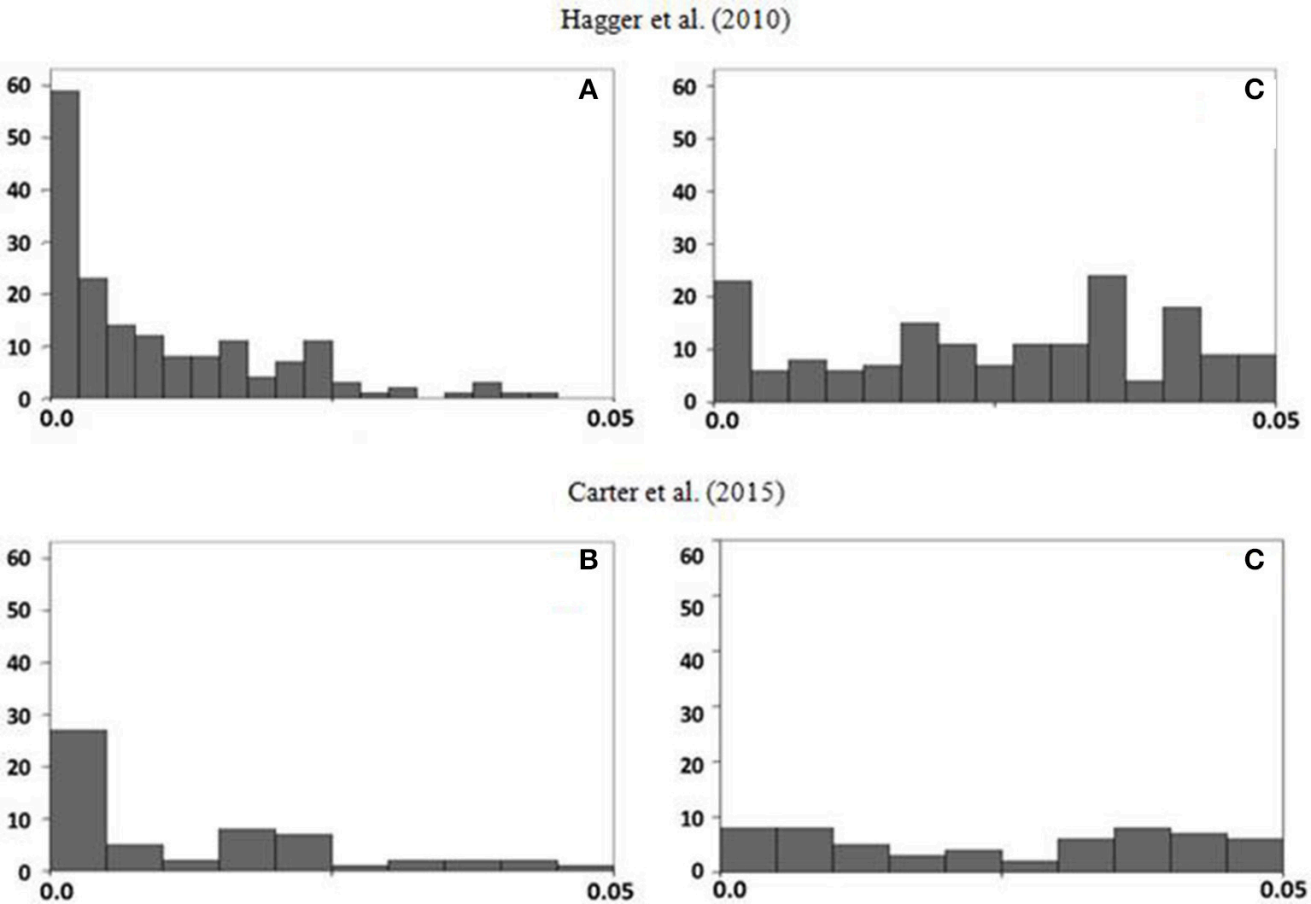
**Initial frequency distributions of ***p***-values for the null hypothesis test (A,B)** and frequency distributions of the delta values of 0.64 and 0.66 (**C,D** respectively).

We have used two methods to estimate the parameter (Van Assen et al., 2015). We applied the *p*-uniform test, with the method-of-the-moments (Irwin-Hall distribution), and the minimal Kolmogorov–Smirnov test, excluding from both datasets every non-significant study as well as those studies in which the interaction was significant but the simple effect was not. As well as the respective authors did before, we truncated the extreme values to a maximum of *d* = 1.5. The R code and the databases from both meta-analysis are available as supplementary materials. The method-of-the-moments provides the values *d* = 0.64 and *d* = 0.66 for the Hagger et al. and the Carter et al. datasets, respectively (Figure 1). The Kolmogorov–Smirnov method yields very similar values: *d* = 0.65 and *d* = 0.66. Therefore, were the *p*-uniform the only method used to analyze both data sets, the naive conclusions would be that: (a) the ego-depletion effect exists, (b) the *ES* is of a moderate magnitude, and (c) the results from both datasets converge. Importantly, the conclusions are not sensitive to the differences between the inclusion/exclusion criteria employed in the two meta-analyses (remember that only 28 of the 116 independent estimates in the Carter et al. dataset were also included by Hagger et al.).

In what follows we elaborate a different interpretation of the evidence, taking into account the results from the *p*-uniform analyses while trying to find some rationale for the incongruences between the results obtained with the different methods and procedures.

In datasets with high publication bias, the *p*-uniform method applied to the significant studies should provide a smaller *ES* estimate than the traditional method applied to all the published studies. However, in datasets with medium to large heterogeneity, the *p*-uniform might yield some overestimate due to the small-studies effect (bias toward large *ES* studies). Then, if we focus only in the *p*-uniform results, our assessment regarding the dataset of Hagger et al. should be that there is no relevant publication bias (the estimates with both methods are very close, and the heterogeneity is low; *I*^2^ = 34.7). The re-analysis made by Carter and McCullough (2014) of Hagger et al.'s dataset, while taking into account the effects of publication bias, provide lower *ES* estimates than those obtained by Hagger et al. However, we know that the asymmetries they found in the funnel plots can be due to different factors than publication bias, and that the regression methods employed are not robust with heterogeneous datasets.

Then, what happens with the dataset of Carter et al.? In that dataset the *p*-uniform method applied to the significant studies yielded an *ES* estimate considerably higher than the traditional method with the complete dataset. One possible explanation is that the strict criteria of inclusion/exclusion employed had important (unintended) collateral consequences on the distribution between the counts of studies with significant and non-significant results. As a result, their database might have an under-representation of significant studies, and the *ES* of 0.43 with the traditional method increases to 0.66 when analyzed with the *p*-uniform method. Alternatively, the increase in the *p*-uniform estimate could be also due to the high heterogenity in Carter et al.'s dataset.

In Carter et al.'s dataset there is a striking result: the specific variance (representing the random effects factor) is much higher (τ^2^ = 0.16; *I*^2^ = 71.6) than in Hagger et al.'s dataset (τ^2^ = 0.04; *I*^2^ = 34.7). The difference between those values also needs an explanation. Relevant for that difference are the results of the RRR. The aim of the RRR initiative was to provide new evidence for the debate about eliminating the threat of publication bias (Hagger and Chatzisarantis, 2016). The process for RRR requires the use of a rigorous protocol that has the advantage (and the disadvantage) that homogenizes the procedures. The conclusion after summarizing the results of 23 independent replications is that the *ES* is not statistically different from zero for the specific experimental paradigm chosen and the set of conditions in the protocol. As expected, after all the efforts made to homogenize the studies, when the dataset was synthetized with a traditional random effects model, the specific variance was smaller than in the two meta-analyses (τ^2^ = 0.02; *I*^2^ = 36.08). The specific variance of Hagger et al. (0.04) and the RRR (0.02) represent similar percentages because the total variance in both cases are different, and the heterogeneity of the sample sizes was larger in Hagger et al. On the other hand, the specific variance in Carter et al. (0.16) represents a much larger percentage of the total variance.

We can view the specific variance in the RRR as a kind of floor value for the heterogeneity in the research with the experimental paradigm they employed. The results of this RRR allow us to state with greater confidence that, at least for the experimental paradigm chosen, the ego-depletion effect is very small, practically irrelevant. This conclusion can only generalize to studies done with the specific protocol of the RRR. However, several subtle sources of variability not controlled in the RRR protocol are responsible for the τ^2^ = 0.02 value (*I*^2^ = 36.08). Then, why is the specific variance in Carter et al. four times larger than in Hagger et al.?

The *p*-uniform method very efficiently overcomes the publication bias threat, but it also has some weaknesses. The most important appear when the average *p*-value of the significant studies is high (0.025–0.050), and when the database shows high heterogeneity (*I*^2^ > 50; van Aert et al., 2016). An anomalously high proportion of *p*-values close to the significance threshold is a strong cue for *p*-hacking. As the average *p*-values in the databases analyzed here are 0.011 for Hagger et al. (median: 0.007) and 0.012 for Carter et al. (median: 0.007) we dismiss such a scenario. The *I*^2^ value of Carter et al.'s dataset is well above 50, but that of Hagger et al. is below that threshold. Again, we should be more confident with the *p*-uniform estimate based on Hagger et al.'s dataset.

## Conclusions

Our analyses add new arguments to the current debate on the ego-depletion effect, showing that it might have been premature to conclude that it is an experimental or statistical artifact. The ego-depletion effect has been proposed as a much more general phenomenon than the specific situation of the RRR protocol. It is expected to appear in many different circumstances. The two databases re-analyzed here include samples of studies that better represent the variety of experimental paradigms in the literature than the studies in the RRR. A good understanding of the effect with all its subtleties requires both intensive efforts focused on specific but varied paradigms under RRR initiatives, and fair procedures to analyze the databases that arise spontaneously from the laboratories. As long as there is no agreement on what a fair procedure is, we should report the results found with several methods that have different strengths and weaknesses. The range of results obtained will ultimately give us a more complete picture.

## Author contributions

DB: Reviewing the literature, elaborating the data bases, designing R programs for calculating and fitting the results, and assessing the results. JB: Assessing the results, writing the manuscript. MS: Designing R programs for calculating and fitting the results, assessing the results.

### Conflict of interest statement

The authors declare that the research was conducted in the absence of any commercial or financial relationships that could be construed as a potential conflict of interest.
