# Supplementary material for: The Debate on the Ego-Depletion Effect: Evidence from Meta-Analysis with the p-Uniform Method
Source: Front Psychol. 2017 Feb 14;8:197. doi: 10.3389/fpsyg.2017.00197 (PMC5306285; doi:10.3389/fpsyg.2017.00197)
Supplement: Supplementary file 1 [file Table1.PDF]

Table 1. Sensitivity analysis for Hagger et al. (2010) meta-analysis when using p-uniform with only statistically significant studies. Id = Identifier, Study = abbreviated reference (includes the first author, the year of publication, and the study within the paper), ES without = p-uniform Effect Size estimate without the mentioned study, Difference = Difference between the p-uniform Effect Size estimate with the whole sample of statistically significant studies (ES = 0.6439) and the p-uniform Effect Size estimate without the mentioned study.

Id	Study	ES without	Difference
1	Alberts, Martijn, Greb, Merckelbach, & Vries (2007) 1	0.6425	-0.0014
2	Alberts, Martijn, Greb, Merckelbach, & Vries (2007) 2	0.6394	-0.0045
3	Baumeister, Bratslavsky, Muraven & Tice (1998) 1	0.6394	-0.0045
4	Baumeister, Bratslavsky, Muraven & Tice (1998) 2	0.6394	-0.0045
5	Baumeister, Bratslavsky, Muraven & Tice (1998) 3	0.6456	0.0017
6	Baumeister, Bratslavsky, Muraven & Tice (1998) 4	0.6454	0.0015
7	Bray, Ginis, Hicks & Woodgate (2008)	0.647	0.0031
8	Bruyneel, Dewitte, Franses & Dekimpe (2009) 2	0.6467	0.0028
9	Bruyneel, Dewitte, Franses & Dekimpe (2009) 3	0.6432	-0.0007
10	Bruyneel, Dewitte, Franses & Dekimpe (2009) 4	0.6469	0.003
11	Bruyneel, Dewitte, Vohs & Warlop (2006) 1	0.646	0.0021
12	Bruyneel, Dewitte, Vohs & Warlop (2006) 2	0.646	0.0021
13	Bruyneel, Dewitte, Vohs & Warlop (2006) 3	0.6467	0.0028
14	Burkley (2008) 1	0.6475	0.0036
15	Burkley (2008) 2	0.6448	0.0009
16	Burkley (2008) 3	0.6476	0.0037
17	Burkley (2008) 4, strong arguments	0.646	0.0021
18	Ciarocco, Sommer & Baumeister (2001) 1	0.6451	0.0012
19	Ciarocco, Sommer & Baumeister (2001) 2	0.6439	0
20	DeWall, Baumeister, Gailliot, & Maner (2008) 1	0.6412	-0.0027
21	DeWall, Baumeister, Gailliot, & Maner (2008) 2	0.6426	-0.0013
22	DeWall, Baumeister, Gailliot, & Maner (2008) 3 stranger condition only	0.6481	0.0042
23	DeWall, Baumeister, Stillman, & Gailliot (2007) 1	0.6469	0.003
24	DeWall, Baumeister, Stillman, & Gailliot (2007) 2 *	0.6449	0.001
25	DeWall, Baumeister, Stillman, & Gailliot (2007) 3	0.6413	-0.0026
26	DeWall, Baumeister, Stillman, & Gailliot (2007) 4	0.6464	0.0025
27	Fennis, Janssen & Vohs (2009) 1	0.6458	0.0019
28	Fennis, Janssen & Vohs (2009) 2	0.646	0.0021
29	Fennis, Janssen & Vohs (2009) 2a	0.6398	-0.0041
30	Fennis, Janssen & Vohs (2009) 3	0.6445	0.0006
31	Fennis, Janssen & Vohs (2009) 4	0.6394	-0.0045
32	Fennis, Janssen & Vohs (2009) 5	0.6456	0.0017
33	Finkel, Campbell, Brunell, Dalton, Scarbeck & Chartrand (2006) 1	0.6439	0
34	Finkel, Campbell, Brunell, Dalton, Scarbeck & Chartrand (2006) 2	0.6426	-0.0013
35	Finkel, Campbell, Brunell, Dalton, Scarbeck & Chartrand (2006) 3	0.6434	-0.0005
36	Finkel, Campbell, Brunell, Dalton, Scarbeck & Chartrand (2006) 4	0.647	0.0031
37	Finkel, Campbell, Brunell, Dalton, Scarbeck & Chartrand (2006) 5 (mean of 2 effect sizes)	0.6435	-0.0004
38	Finkel, DeWall, Slotter, Oaten & Foshee (in press)	0.641	-0.0029
39	Fischer, Greitemeyer & Frey (2007) 1, mean effect across 4 ability ratings	0.6441	0.0002
40	Fischer, Greitemeyer & Frey (2007) 2	0.6473	0.0034

41	Fischer, Greitemeyer & Frey (2007) 3, mean prob of serious disease & optimism	0.6456	0.0017
42	Fischer, Greitemeyer & Frey (2007) 4, main effect for ego depletion across +ibe & -ive attri	0.6458	0.0019
43	Fischer, Greitemeyer & Frey (2007) 5, above average effect	0.6454	0.0015
44	Fischer, Greitemeyer & Frey (2008) 1	0.646	0.0021
45	Fischer, Greitemeyer & Frey (2008) 2	0.6451	0.0012
46	Fischer, Greitemeyer & Frey (2008) 3	0.6435	-0.0004
47	Fischer, Greitemeyer & Frey (2008) 4	0.6422	-0.0017
48	Gailliot & Baumeister (2007) 2	0.6426	-0.0013
49	Gailliot & Baumeister (2007) 3	0.6449	0.001
50	Gailliot, Baumeister, DeWall, Maner, Plant, Tice, Brewer & Schmeichel (2007), 7	0.6435	-0.0004
51	Gailliot, Baumeister, DeWall, Maner, Plant, Tice, Brewer & Schmeichel (2007), 8 (using placebo condition only)	0.6476	0.0037
52	Gailliot, Plant, Butz, & Baumeister (2007) 1	0.6424	-0.0015
53	Gailliot, Plant, Butz, & Baumeister (2007) 2	0.6479	0.004
54	Gailliot, Plant, Butz, & Baumeister (2007) 3	0.648	0.0041
55	Gailliot, Schmeichel & Baumeister (2006) 2	0.6444	0.0005
56	Gailliot, Schmeichel & Baumeister (2006) 3	0.6473	0.0034
57	Gailliot, Schmeichel & Baumeister (2006) 6	0.6461	0.0022
58	Gailliot, Schmeichel & Baumeister (2006) 7 S1	0.647	0.0031
59	Gailliot, Schmeichel & Baumeister (2006) 8	0.6456	0.0017
60	Gailliot, Schmeichel & Baumeister (2006) 9 (neutral word completion puzzles only)	0.6443	0.0004
61	Gailliot, Schmeichel & Maner (2007) 1	0.6394	-0.0045
62	Geeraert & Yzerbyt (2007) 1b	0.6479	0.004
63	Geeraert & Yzerbyt (2007) 2b	0.6408	-0.0031
64	Gordijin, Hindricks, Koomen, Dijksterhuis, & Knippenberg (2004) 2	0.6459	0.002
65	Gordijin, Hindricks, Koomen, Dijksterhuis, & Knippenberg (2004) 4 low suppres motiv	0.6433	-0.0006
66	Govorun & Payne (2006)	0.6476	0.0037
67	Inzlicht & Gutsell (2007)	0.6394	-0.0045
68	Inzlicht, McKay, & Aronson (2006) 2	0.6429	-0.001
69	Inzlicht, McKay, & Aronson (2006) 3	0.6447	0.0008
70	Janssen, Fennis, Pruyn, & Vohs (2008) 2	0.64	-0.0039
71	Johns, Inzlicht, & Schmader 1 (2008)	0.6436	-0.0003
72	Johns, Inzlicht, & Schmader 3 (2008)	0.648	0.0041
73	Johns, Inzlicht, & Schmader 4 (2008)	0.6414	-0.0025
74	Kahan, Polivy & Herman (2003)	0.647	0.0031
75	Legault, Green-Demers, & Eadie (2009)	0.6459	0.002
76	Martijn, Alberts, Merckelbach, Havermans, Huijts, Vries (2007)	0.6457	0.0018
77	Martijn, Tenbult, Merckelbach, Dreezens, & de Vries (2002)	0.6458	0.0019
78	Mead et al. (2009) 1	0.6455	0.0016
79	Mead et al. (2009) 2	0.6407	-0.0032
80	Moller, Deci, & Ryan (2006) 1	0.6475	0.0036
81	Muraven & Slessareva (2003) 1	0.6468	0.0029
82	Muraven & Slessareva (2003) 2	0.6466	0.0027
83	Muraven (2008, JASP) 1	0.6419	-0.002

84	Muraven (2008, JASP) 2	0.6459	0.002
85	Muraven, Collins, & Nienhaus (2002)	0.647	0.0031
86	Muraven, Gagne, & Rosman (2008) 1 (OUTLIER! d=3.02, set at d=1.90)	0.6395	-0.0044
87	Muraven, Gagne, & Rosman (2008) 3	0.6399	-0.004
88	Muraven, Shmueli, Burkley (2006) 1	0.6467	0.0028
89	Muraven, Shmueli, Burkley (2006) 4	0.6455	0.0016
90	Muraven, Tice & Baumeister (1998) 1	0.6451	0.0012
91	Muraven, Tice & Baumeister (1998) 2	0.6452	0.0013
92	Muraven, Tice & Baumeister (1998) 3	0.6469	0.003
93	Neshat-Doost, Dagleish & Golden (2008)	0.644	0.0001
94	Oaten, Williams, Jones, & Zadro (2008) 1	0.6397	-0.0042
95	Oaten, Williams, Jones, & Zadro (2008) 2	0.6456	0.0017
96	Oikawa (2005) 1	0.6415	-0.0024
97	Oikawa (2005) 2	0.6394	-0.0045
98	Ostafin, Marlatt & Greenwald (2008)	0.6476	0.0037
99	Park, Glaser & Knowles (2008)	0.6466	0.0027
100	Pocheptsova et al. (2009) 1	0.6484	0.0045
101	Pocheptsova et al. (2009) 2	0.6484	0.0045
102	Richeson & Shelton (2003) [N is exp group, control group]	0.6401	-0.0038
103	Richeson & Trawalter (2005) 1	0.6426	-0.0013
104	Richeson & Trawalter (2005) 2	0.6427	-0.0012
105	Richeson & Trawalter (2005) 3	0.6427	-0.0012
106	Richeson, Trawalter, & Shelton (2005)	0.6427	-0.0012
107	Schmeichel & Vohs (2009) 1	0.6457	0.0018
108	Schmeichel & Vohs (2009) 2	0.6428	-0.0011
109	Schmeichel (2007) 1 sample 1 operation span (average of measures)	0.6476	0.0037
110	Schmeichel (2007) 1 sample 2 sentence span (average of measures)	0.647	0.0031
111	Schmeichel (2007) 2 sample 1 forward span (total measure)	0.6472	0.0033
112	Schmeichel (2007) 3 STM-2 vs. working memory task	0.647	0.0031
113	Schmeichel (2007) 4 (average of measures)	0.6468	0.0029
114	Schmeichel, Demaree, Robinson & Pu (2006) (mean es across verb/figural)	0.6422	-0.0017
115	Schmeichel, Vohs & Baumeister (2003) 1 (no. GRE correct)	0.6396	-0.0043
116	Schmeichel, Vohs & Baumeister (2003) 3	0.6426	-0.0013
117	Seeley & Gardner (2003) 1	0.6482	0.0043
118	Seeley & Gardner (2003) 2	0.6481	0.0042
119	Segerstrom & Solberg Nes (2007) - persistence 1st unsolveable anagram	0.6443	0.0004
120	Shamosh & Gray (2007)	0.6447	0.0008
121	Stucke & Baumeister (2006) 1	0.6422	-0.0017
122	Stucke & Baumeister (2006) 2	0.6457	0.0018
123	Stucke & Baumeister (2006) 3	0.6398	-0.0041
124	Tice, Baumeister, Shmueli, & Muraven (2007) 2	0.6453	0.0014
125	Trawalter & Richeson (2006)	0.6432	-0.0007
126	Tyler & Burns (2008) 1	0.6394	-0.0045
127	Tyler & Burns (2008) 2	0.6413	-0.0026
128	Tyler & Burns (2009) 1	0.6406	-0.0033
129	Tyler & Burns (2009) 2 S1	0.6415	-0.0024

130	Tyler & Burns (2009) 2 S2 (OUTLIER! d=2.60, set at d=1.90)	0.6394	-0.0045
131	Tyler & Burns (2009) 3 not finished condition only	0.64	-0.0039
132	Tyler (2008) 1	0.6411	-0.0028
133	Tyler (2008) 2	0.6424	-0.0015
134	Tyler (2008) 3	0.6398	-0.0041
135	Tyler (2008) 4	0.6461	0.0022
136	Vohs & Faber (2007) 1	0.642	-0.0019
137	Vohs & Faber (2007) 2	0.6394	-0.0045
138	Vohs & Faber (2007) 3	0.6395	-0.0044
139	Vohs & Heatherton (2000) 1	0.6406	-0.0033
140	Vohs & Heatherton (2000) 2	0.6458	0.0019
141	Vohs & Heatherton (2000) 3	0.6448	0.0008
142	Vohs & Schmeichel (2003) 3	0.6402	-0.0037
143	Vohs & Schmeichel (2003) 4	0.6438	-0.0001
144	Vohs, Baumeister & Ciarocco (2005) 1	0.6459	0.002
145	Vohs, Baumeister & Ciarocco (2005) 2	0.6452	0.0013
146	Vohs, Baumeister & Ciarocco (2005) 3	0.6395	-0.0044
147	Vohs, Baumeister & Ciarocco (2005) 4	0.64	-0.0039
148	Vohs, Baumeister & Ciarocco (2005) 5	0.6443	0.0004
149	Vohs, Baumeister & Ciarocco (2005) 6 (mean of avoidant and ambivalent only)	0.6454	0.0015
150	Vohs, Baumeister & Ciarocco (2005) 7	0.6437	-0.0002
151	Vohs, Baumeister & Ciarocco (2005) 8	0.6448	0.0009
152	Vohs, Baumeister, Schmeichel, Twenge, Nelson, & Tice (2008) 1a	0.6398	-0.0041
153	Vohs, Baumeister, Schmeichel, Twenge, Nelson, & Tice (2008) 1b	0.642	-0.0019
154	Vohs, Baumeister, Schmeichel, Twenge, Nelson, & Tice (2008) 2	0.6431	-0.0008
155	Vohs, Baumeister, Schmeichel, Twenge, Nelson, & Tice (2008) 3	0.6428	-0.0011
156	Vohs, Baumeister, Schmeichel, Twenge, Nelson, & Tice (2008) 4a	0.6431	-0.0008
157	Vohs, Baumeister, Schmeichel, Twenge, Nelson, & Tice (2008) 4b	0.6419	-0.002
158	Vohs, Baumeister, Schmeichel, Twenge, Nelson, & Tice (2008) 6	0.6435	-0.0004
159	Wallace & Baumeister (2002)	0.6418	-0.0021
160	Wan & Sternthal (2008) 1	0.6406	-0.0033
161	Wan & Sternthal (2008) 2 no clock	0.6403	-0.0036
162	Wan & Sternthal (2008) 3	0.6434	-0.0005
163	Wan & Sternthal (2008) 4	0.6403	-0.0036
164	Webb & Sheeran (2003) 1	0.6426	-0.0013
165	Webb & Sheeran (2003) 2	0.6394	-0.0045
166	Wheeler, Brinol & Hermann (2007)	0.6409	-0.003
167	Wright , Stewart & Barnett (2008) 2 S2	0.6452	0.0013
168	Zyphur, Warren, Landis & Thoresen (2007) 1 (mean of candy&puzzle)	0.6447	0.0008
169	Zyphur, Warren, Landis & Thoresen (2007) 2	0.646	0.0021
