# Supplementary material for: The Debate on the Ego-Depletion Effect: Evidence from Meta-Analysis with the p-Uniform Method
Source: Front Psychol. 2017 Feb 14;8:197. doi: 10.3389/fpsyg.2017.00197 (PMC5306285; doi:10.3389/fpsyg.2017.00197)
Supplement: Supplementary file 2 [file Table2.PDF]

Table 2. Sensitivity analysis for Carter et al. (2015) meta-analysis when using p-uniform with only statistically significant studies. Id = Identifier, Study = abbreviated reference (includes the first author, the study within the paper, and the year of publication), ES without = p-uniform Effect Size estimate without the mentioned study, Difference = Difference between the p-uniform Effect Size estimate with the whole sample of statistically significant studies (ES = 0.67) and the p-uniform Effect Size estimate without the mentioned study.

Id	Study	ES without	Difference
1	BaumeisterB 1 1998	0.65478046	-0.0152664
2	BaumeisterB 3 1998	0.67739447	0.00734761
3	BaumeisterD 2 2005	0.66659464	-0.00345222
4	BaumeisterD 3 2005	0.66133023	-0.00871663
5	BoucherK 1 2012	0.68205452	0.01200766
6	BoucherK 2 2012	0.66854379	-0.00150307
7	ChristiansenC 0 2012	0.67223068	0.00218382
8	ClarksonH 1 2010	0.67525817	0.00521131
9	DeWallB 3 2008b	0.66263183	-0.00741503
10	DvorakS 0 2009	0.65670584	-0.01334102
11	EganH 1 2012	0.67708157	0.00703471
12	EganH 2 2012	0.65677409	-0.01327277
13	GailliotB 7 2007c	0.68369305	0.01364619
14	GeeraertC 1 2013	0.6635753	-0.00647156
15	GeeraertC 2 2013	0.68308928	0.01304242
16	GeeraertY 1b 2007	0.68424987	0.01420301
17	Gohar 3 2011	0.67603552	0.00598866
18	HealeyH 1 2011	0.65592193	-0.01412493
19	HealeyH 3 2011	0.67660793	0.00656107
20	ImhoffS 1 2013	0.66868845	-0.00135841
21	InzlichtG 0 2007	0.68173005	0.01168319
22	MartijnT 1 2002	0.67822225	0.00817539
23	MasicampoR 5 2011	0.66956505	-0.00048181
24	MoldenD 2 2012	0.66680478	-0.00324208
25	MuravenS 1 2003	0.68193789	0.01189103
26	MuravenS 4 2005	0.65990829	-0.01013857
27	MuravenS 4 2005	0.67061315	0.00056629
28	MuravenT 1 1998	0.67427656	0.0042297
29	MuravenT 2 1998	0.66291843	-0.00712843
30	OatenW 1 2008	0.6546143	-0.01543256
31	PondD 3 2011	0.68476508	0.01471822
32	Ruci 2 2003	0.67782864	0.00778178
33	ScherschelM 1 2011	0.68494786	0.014901
34	Schmeichel 1a 2007	0.68302442	0.01297756
35	Schmeichel 1b 2007	0.68214508	0.01209822
36	Schmeichel 2 2007	0.6818155	0.01176864
37	Schmeichel 3 2005	0.67899649	0.00894963
38	Schmeichel 4 2007	0.68049579	0.01044893
39	SchmeichelV 1 2003	0.65849711	-0.01154975
40	SeeleyG 2 2003 TS	0.66720715	-0.00283971
41	Smith 1 2002	0.65470654	-0.01534032
42	Smith p1 2002	0.65657386	-0.013473
43	TylerB 2 2009	0.65549865	-0.01454821
44	TylerB 3 2009	0.65795851	-0.01208835
45	UzielL 3 2012	0.68093501	0.01088815
46	VohsB 5 2013	0.67853749	0.00849063
47	VohsH 2 2000	0.67513002	0.00508316
48	VohsH 3 2000	0.67526239	0.00521553
49	WallaceB 0 2002	0.66520434	-0.00484252
50	Wan 1 2007	0.65894977	-0.01109709
51	Wan 2 2007	0.65829053	-0.01175633

52	Wan 3 2007	0.6632022	-0.00684466
53	Wan 4 2007	0.65783174	-0.01221512
54	Wan 6 2007	0.65829053	-0.01175633
55	Wan 7 2007	0.67417823	0.00413137
56	Wan 8 2007	0.6591468	-0.01090006
57	XuH 0 2012	0.67662477	0.00657791
