# Supplementary material for: The Debate on the Ego-Depletion Effect: Evidence from Meta-Analysis with the p-Uniform Method
Source: Front Psychol. 2017 Feb 14;8:197. doi: 10.3389/fpsyg.2017.00197 (PMC5306285; doi:10.3389/fpsyg.2017.00197)
Supplement: Supplementary file 7 [file DataSheet5.DOCX]

Okay, now we are getting somewhere! Thank you for clarifying that "studies" and "effects" are

used interchangeably.

Can you explain how you submitted non-significant p-values to p-curve? As of 4.0, the online

app automatically discards all non-significant p-values. Do you have a different p-curve

application or analysis program?

Instead of using the online app, we developed a program with R syntax that allow us to

employ two methods developed within the ‘significant only’ perspective. According to the

specifications given by Van Assen et al. (2015), this new program allow us to obtain estimates

of the parameter δ by using the method-of-the-moments and the Kolmogorov-Smirnov test.

In the method-of-the-moments when the p-values are normalized for the true population

parameter the expected value for its sum is k• (1/2) (Irwin-Hall distribution). Our estimate is

the value for which that sum equals the expected value. In the method based on the test of

Kolmogorov-Smirnov, the test is applied to assess the fit of the empirical frequencies of the

normalized p-values to a uniform distribution. Our estimate is the value that minimizes the

Kolmogorov-Smirnov statistic.

Okay, now we are getting somewhere! Thank you for clarifying that "studies" and "effects" are

used interchangeably.

Can you explain how you submitted non-significant p-values to p-curve? As of 4.0, the online

app automatically discards all non-significant p-values. Do you have a different p-curve

application or analysis program?

Instead of using the online app, we developed a program with R syntax that allow us to

employ two methods developed within the ‘significant only’ perspective. According to the

specifications given by Van Assen et al. (2015), this new program allow us to obtain estimates

of the parameter δ by using the method-of-the-moments and the Kolmogorov-Smirnov test.

In the method-of-the-moments when the p-values are normalized for the true population

parameter the expected value for its sum is k• (1/2) (Irwin-Hall distribution). Our estimate is

the value for which that sum equals the expected value. In the method based on the test of

Kolmogorov-Smirnov, the test is applied to assess the fit of the empirical frequencies of the

normalized p-values to a uniform distribution. Our estimate is the value that minimizes the

Kolmogorov-Smirnov statistic.

we developed a program with R syntax that allow us to

employ two methods developed within the ‘significant only’ perspective. According to the

specifications given by Van Assen et al. (2015), this new program allow us to obtain estimates

of the parameter δ by using the method-of-the-moments and the Kolmogorov-Smirnov test.

In the method-of-the-moments when the p-values are normalized for the true population

parameter the expected value for its sum is k• (1/2) (Irwin-Hall distribution). Our estimate is

the value for which that sum equals the expected value. In the method based on the test of

Kolmogorov-Smirnov, the test is applied to assess the fit of the empirical frequencies of the

normalized p-values to a uniform distribution. Our estimate is the value that minimizes the

Kolmogorov-Smirnov statistic.

we developed a program with R syntax that allow us to

employ two methods developed within the ‘significant only’ perspective. According to the

specifications given by Van Assen et al. (2015), this new program allow us to obtain estimates

of the parameter δ by using the method-of-the-moments and the Kolmogorov-Smirnov test.

In the method-of-the-moments when the p-values are normalized for the true population

parameter the expected value for its sum is k• (1/2) (Irwin-Hall distribution). Our estimate is

the value for which that sum equals the expected value. In the method based on the test of

Kolmogorov-Smirnov, the test is applied to assess the fit of the empirical frequencies of the

normalized p-values to a uniform distribution. Our estimate is the value that minimizes the

Kolmogorov-Smirnov statistic.

#This R code obtains an estimate for the parameter delta from the use of uniroot function and two

#methods:

#a) The method-of-the-moments with the Irwin-Hall distribution

#b) The Kolmogorov-Smirnov test

#This program can obtain an estimate for the parameter delta from the use of the stadistically significant

#primary studies as well as the whole sample of published studies (statistically significant and non-significant

#results).

#OBSERVATIONS Calculations are designed for a right tailed test and a level of significance of 0.05.

#DATA RETRIEVAL The input is a matrix data with the rows representing the primary studies. The first

#column representing an abbreviated reference for each primary study, the second colum representing

#the effect size measure Cohen’s d, the third column representing the sample size for the experimental

#group, the fourth column representing the sample size for the control group, the fifth colum representing

#the standard deviation for the effect size, the sixth colum representing the test statistic, the seventh

#colum representing the p-value when testing the null hypothesis, and the eighth colum representing the

#statistical significance (0 = statistically non-significant result, 1 = statistically significant result).

#The matrix data should be entered via a CSV file with a comma as separator and a period indicating the

#decimal point.

#P-UNIFORM RESULTS The estimates of the parameter delta will appear organized in the next matrix by the

#meta-analytic study (Hagger et al. (2010) or Carter et al. (2015)), by the method used (Irwin-Hall

#distribution and the method of moments, or the Kolmogorov-Smirnov test), and by the sample of studies

#used (statistically significant results or all published results).

puniform_results <- matrix(NA, nrow = 5, ncol = 3)

puniform_results[1,2] <- c("Hagger et al.(2010)")

puniform_results[1,3] <- c("Carter et al.(2015)")

puniform_results[2,1] <- c("Irwin-Hall (significant results)")

puniform_results[3,1] <- c("Irwin-Hall (all results)")

puniform_results[4,1] <- c("Kolmogorov-Smirnov (significant results)")

puniform_results[5,1] <- c("Kolmogorov-Smirnov (all results)")

#Setting the working directory where the data of the primary studies is gotten.

setwd("C:/Users/USUARIO/Desktop/Meta-analysis")

#IRWIN-HALL SIGNIFICANT STUDIES This first program section obtains the estimate of the parameter by

#using the method-of-the-moments and the Irwin-Hall distribution with only the statistically significant

#primary studies. “IrHal_fun_sig_only” is the function defined for uniroot to estimate the parameter delta

#(“delta_estimate”).

#Getting the primary studies with statistically significant results. Here, we have chosen the significant

#primary studies of Hagger et al. (2010) to begin with.

sig_studies <- read.csv("Hagger2010_sig_studies.csv ", header = TRUE, sep = ";")

#The vector named:

#“d” collects the effect size measures.

#“n1” collects the sample sizes for the control group.

#“n2” collects the sample sizes for the experimental group.

#“sd” represents the standard deviation of Cohen’s d.

#“critical_d” obtains the critical d value for each study (the value under which the effect would result

#satistically non-significant given the study’s sample size). If any other level of significance is needed (see

#OBSERVATIONS) the value 1.645 should be changed accordingly.

#“sd_critical_d” estimates the standard deviation of the critical d value.

d <- sig_studies[,2]

n1 <- sig_studies[,3]

n2 <- sig_studies[,4]

sd <- sqrt( (n1 + n2) / (n1 * n2) )

critical_d <- 1.645 * sd

sd_critical_d <- sqrt( (n1 + n2) / (n1 * n2) )

IrHal_fun_sig_only <- function(vector_d, vector_critical_d, vector_sd_d, vector_sd_critical_d, valo, delta_estim)

{

vector_Z <- (vector_d - delta_estim) / vector_sd_d

vector_C <- (vector_critical_d - delta_estim) / vector_sd_critical_d

valo - sum((pnorm(vector_Z, lower.tail=FALSE) / pnorm(vector_C, lower.tail=FALSE)))

}

#RESULTS The estimate of the parameter delta for Hagger et al. (2010) when using only the statistically

#significant results is the object root within the list “output” given by the uniroot function.

output <- uniroot(IrHal_fun_sig_only, c(-2,2), vector_d = d, vector_critical_d = critical_d, vector_sd_d = sd, vector_sd_critical_d = sd_critical_d, valo = length(d) / 2, maxiter = 10000, trace = 3)

Hagger_IH_sig_delta_estimate <- output$root

puniform_results[2,2] <- Hagger_IH_sig_delta_estimate

#The estimate of the parameter delta for Carter et al. (2015) when using only the statistically significant

#results is gotten the same way.

sig_studies <- read.csv("Carter2015_sig_studies.csv ", header = TRUE, sep = ";")

d <- sig_studies[,2]

n1 <- sig_studies[,3]

n2 <- sig_studies[,4]

sd <- sqrt( (n1 + n2) / (n1 * n2) )

critical_d <- 1.645 * sd

sd_critical_d <- sqrt( (n1 + n2) / (n1 * n2) )

output <- uniroot(IrHal_fun_sig_only, c(-2,2), vector_d = d, vector_critical_d = critical_d, vector_sd_d = sd, vector_sd_critical_d = sd_critical_d, valo= length(d) / 2, maxiter=10000, trace=3)

Carter_IH_sig_delta_estimate <- output$root

puniform_results[2,3] <- Carter_IH_sig_delta_estimate

#IRWIN-HALL ALL STUDIES This second program section obtains the estimate of the parameter by using the

#method-of-the-moments and the Irwin-Hall distribution with the whole sample of primary studies

#(statistically significant and non-significant results). “IrHal_fun” is the function defined for uniroot to

#estimate the parameter delta “delta_estimate”.

#Getting the whole sample of primary studies (statistically significant and non-significant results). Here, we #have chosen the whole sample of primary studies of Hagger et al. (2010) to begin with.

all_studies <- read.csv("Hagger2010_all_studies.csv ", header = TRUE, sep = ";")

#The vector named:

#“d” collects the effect size measures.

#“n1” collects the sample sizes for the control group.

#“n2” collects the sample sizes for the experimental group.

#“sd” represents the standard deviation of Cohen’s d.

d <- all_studies [,2]

n1 <- all_studies [,3]

n2 <- all_studies [,4]

sd <- sqrt( (n1 + n2) / (n1 * n2))

IrHal_fun <- function(vector_d, vector_sd_d, valo, delta_estim)

{

vector_Z <- (vector_d - delta_estim) / vector_sd_d

valo - sum((pnorm(vector_Z, lower.tail=FALSE)))

}

#RESULTS The estimate of the parameter delta for Hagger et al. (2010) when using the whole sample of

#primary studies (statistically significant and non-significant results) is the object root within the list “output”

#given by the uniroot function.

output <- uniroot(IrHal_fun, c(-2,2), vector_d = d, vector_sd_d = sd, valo= length(d)/2, maxiter=10000, trace=3)

Hagger_IH_all_delta_estimate <- output$root

puniform_results[3,2] <- Hagger_IH_all_delta_estimate

#The estimate of the parameter delta for Carter et al. (2015) when using the whole sample of primary

#studies (statistically significant and non-significant results) is gotten the same way.

all_studies <- read.csv("Carter2015_all_studies.csv ", header = TRUE, sep = ";")

d <- all_studies [,2]

n1 <- all_studies [,3]

n2 <- all_studies [,4]

sd <- sqrt( (n1 + n2) / (n1 * n2) )

output <- uniroot(IrHal_fun, c(-2,2), vector_d = d, vector_sd_d = sd, valo= length(d)/2, maxiter=10000, trace=3)

Carter_IH_all_delta_estimate <- output$root

puniform_results[3,3] <- Carter_IH_all_delta_estimate

#KOLMOGOROV-SMIRNOV SIGNIFICANT STUDIES This third program section estimates the parameter by

#using the Kolmogorov-Smirnov test with only the statistically significant primary studies. The program

#estimates the value for the parameter that, when tested, the Kolmogorov-Smirnov test statistics asserts the

#best fit to a uniform distribution of the frequencies of the resulting p-values . “KS_fun_sig_only” is the

#function defined to estimate the parameter delta “delta_estim” when optimized for its minimum.

# Getting the primary studies with statistically significant results. Here, we have chosen the significant

#primary studies of Hagger et al. (2010) to begin with.

sig_studies <- read.csv("Hagger2010_sig_studies.csv ", header = TRUE, sep = ";")

# The vector named:

# “d” collects the effect size measures.

# “n1” collects the sample sizes for the control group.

# “n2” collects the sample sizes for the experimental group.

# “sd” represents the standard deviation of Cohen’s d.

# “critical_d” obtains the critical d value for each study (the value under which the effect would result

# satistically non-significant given the study’s sample size). If any other level of significance is needed (see

# OBSERVATIONS) the value 1.645 should be changed accordingly.

# “sd_critical_d” estimates the standard deviation of the critical d value.

d <- sig_studies[,2]

n1 <- sig_studies[,3]

n2 <- sig_studies[,4]

sd <- sqrt( (n1 + n2) / (n1 * n2) )

critical_d <- 1.645 * sd

sd_critical_d <- sqrt( (n1 + n2) / (n1 * n2) )

KS_fun_sig_only <- function(delta_estim, vector_d, vector_sd, vector_n1, vector_n2, vector_critical_d, vector_sd_critical_d)

{

p_value <- pt((vector_d - delta_estim) / vector_sd, df = vector_n1 + vector_n2 - 2, lower.tail = F)

p_max <- pt((vector_critical_d - delta_estim) / vector_sd_critical_d, df = vector_n1 + vector_n2 - 2, lower.tail = F)

p_norm <- p_value / p_max

KS_result <- ks.test(p_norm, "punif")

KS_result$statistic

}

#RESULTS The estimate of the parameter delta for Hagger et al. (2010) when using only the statistically

#significant results is the delta value that minimizes the Kolmogorov-Smirnov statistic.

output <- optimize(KS_fun_sig_only, c(-2, 2), vector_d = d, vector_sd = sd, vector_n1 = n1, vector_n2 = n2, vector_critical_d = critical_d, vector_sd_critical_d = sd_critical_d)

Hagger_KS_sig_delta_estimate <- output$minimum

puniform_results[4,2] <- Hagger_KS_sig_delta_estimate

#The estimate of the parameter delta for Carter et al. (2015) when using only the statistically significant

#results is gotten the same way.

sig_studies <- read.csv("Carter2015_sig_studies.csv ", header = TRUE, sep = ";")

d <- sig_studies[,2]

n1 <- sig_studies[,3]

n2 <- sig_studies[,4]

sd <- sqrt( (n1 + n2) / (n1 * n2) )

critical_d <- 1.645 * sd

sd_critical_d <- sqrt( (n1 + n2) / (n1 * n2) )

output <- optimize(KS_fun_sig_only, c(-2, 2), vector_d = d, vector_sd = sd, vector_n1 = n1, vector_n2 = n2, vector_critical_d = critical_d, vector_sd_critical_d = sd_critical_d)

Carter_KS_sig_delta_estimate <- output$minimum

puniform_results[4,3] <- Carter_KS_sig_delta_estimate

#KOLMOGOROV-SMIRNOV ALL STUDIES This fourth program section estimates the parameter by using the

#Kolmogorov-Smirnov test with the whole sample of primary studies (statistically significant and non-

#significant results). The program estimates the value for the parameter that, when tested, the Kolmogorov-

#Smirnov test statistics asserts the best fit to a uniform distribution of the frequencies of the resulting

#p-values . “KS_fun” is the function defined to estimate the parameter delta “delta_estim” when optimized

#for its minimum.

# Getting the whole sample of primary studies (statistically significant and non-significant results). Here, we

#have chosen the whole sample of primary studies of Hagger et al. (2010) to begin with.

all_studies <- read.csv("Hagger2010_all_studies.csv ", header = TRUE, sep = ";")

# The vector named:

# “d” collects the effect size measures.

# “n1” collects the sample sizes for the control group.

# “n2” collects the sample sizes for the experimental group.

# “sd” represents the standard deviation of Cohen’s d.

d <- all_studies[,2]

n1 <- all_studies[,3]

n2 <- all_studies[,4]

sd <- sqrt( (n1 + n2) / (n1 * n2) )

KS_fun <- function(delta_estim, vector_d, vector_sd, vector_n1, vector_n2)

{

p_value <- pt((vector_d - delta_estim) / vector_sd, df = vector_n1 + vector_n2 - 2, lower.tail = F)

KS_result <- ks.test(p_value, "punif")

KS_result$statistic

}

#RESULTS The estimate of the parameter delta for Hagger et al. (2010) when using the whole sample of

#primary studies (statistically significant and non-significant results) is the delta value that minimizes the

#Kolmogorov-Smirnov statistic.

output <- optimize(KS_fun, c(-2, 2), vector_d = d, vector_sd = sd, vector_n1 = n1, vector_n2 = n2)

Hagger_KS_all_delta_estimate <- output$minimum

puniform_results[5,2] <- Hagger_KS_all_delta_estimate

#The estimate of the parameter delta for Carter et al. (2015) when using the whole sample of primary

#studies (statistically significant and non-significant results) is gotten the same way.

all_studies <- read.csv("Carter2015_all_studies.csv ", header = TRUE, sep = ";")

d <- all_studies[,2]

n1 <- all_studies[,3]

n2 <- all_studies[,4]

sd <- sqrt( (n1 + n2) / (n1 * n2) )

output <- optimize(KS_fun, c(-2, 2), vector_d = d, vector_sd = sd, vector_n1 = n1, vector_n2 = n2)

Carter_KS_all_delta_estimate <- output$minimum

puniform_results[5,3] <- Carter_KS_all_delta_estimate
